# Case of Transposition of the Viscera of the Thorax and Abdomen

**Published:** 1826-10

**Authors:** 

**Affiliations:** St. George's Hospital


### TRANSPOSITION OF THE VISCERA.

### *Case of Transposition of the Viscera of the Thorax and Abdomen, in a Patient treated at* St. George's Hospital, by Mr. Rose.

Mary Randall, forty years of age, was admitted into St. George's Hospital, under the care of Mr. Rose, on the 10th of August, 1825, with disease of her right ankle. She had been ill two years, and during the greater part of that time she had been confined to bed. Several ulcerated openings on her instep, and round her ankle, led down to diseased bone: she felt intense pain on the slightest attempt to move her foot; was much emaciated, and beginning to suffer from hectic fever.

On the 19th of August, Mr. Rose amputated the limb below the knee. On examination, the disease was found to have begun in ulceration of the cartilages between the astragalus and os calcis. The stump healed favourably; but she continued in the hospital on account of a large ulcer, the consequence of a slough of the integuments over the sacrum. When it was nearly well, she was attacked with disease of the lungs; of which she died on the 29th of January, 1826.

The body was examined on the following day, and the lungs on both sides were found to be filled with tubercles, many of which had gone into suppuration.

This woman afforded an instance of a complete transposition of the viscera, both of the thorax and abdomen.

The lungs of the right side were divided into two lobes; those of the left into three. The apex of the heart was turned towards the right. The aorta was given off by the right ventricle, which was the most muscular; and the pulmonary artery by the left. The distribution of the veins corresponded; the left auricle receiving the blood from the two venæ cavæ, and the right from the pulmonary veins. The arch of the aorta was turned from the left to the right, and the first great branch given off by it was the common trunk of the carotid and subclavian of the left; the next, the carotid of the right; and the third, the subclavian of the right. The aorta descended to the right side of the spine; and, in the abdomen, had the cava ascendens placed on its left.

The viscera of the abdomen were transposed in a similar manner. The liver was situated in the left hypochondriac region, with its small lobe to the right, and its great lobe to the left side. The stomach and spleen were on the right. The mesentery began on the right side. The ilium terminated in the cœcum on the left side; the transverse arch of the colon passed from the left to the right; and the sigmoid flexure of the colon descended on the right to its termination in the rectum.

Many instances of this *lusus naturæ* are now upon record. One was met with in the dissecting-room of Windmill-street, in 1788; of which an accurate description is given by the late Dr. Baillie, in the Transactions of the Royal Society. Another is mentioned by M. Larrey,* in his Memoirs of Military Surgery.

In examining the case which we have above detailed, Mr. Rose stated that a transposition of the viscera had occurred in a soldier of the Grenadier Guards, whose body was examined at Chatham, by Mr. Bacot; and that he himself had met with another instance in a soldier of the Coldstream Guards, who died at St. Jean de Luz, in the south of France, in 1814; which was seen by Sir James M'Grigor, and by many of the military surgeons. In the latter case, there was no transverse arch of the colon. The cœcum was placed under the left kidney, and, as well as the ascending portion of the colon, was attached, by a loose reflection of peritoneum, to the psoas magnus of the left side. The colon having ascended on the left, and reached the left hypochondrium under the great lobe of the liver which occupied that region, was reflected outwards, and descended on the same side, instead of crossing the epigastric region. The sigmoid flexure of the colon was thus placed on its proper side, (the left,) immediately contiguous to the ascending portion, and on its outer edge. The transposition of the viscera of the thorax, and of all the other viscera of the abdomen, was complete, and precisely similar to that in the case of Randall.

* Vide Mémoires de Chirurgie Militaire, par M. Larrey, tome i. fol. 7.

